# Editorial: Are natural products, used as antitumoral/antiangiogenic agents, less toxic than synthetic conventional chemotherapy?

**DOI:** 10.3389/fphar.2022.1055516

**Published:** 2022-10-21

**Authors:** Cecilia Veronica Nunez, Marne Carvalho de Vasconcellos, Laura Alaniz

**Affiliations:** ^1^ Bioprospection and Biotechnology Laboratory, National Institute of Amazonian Research (INPA), Manaus, Brazil; ^2^ Faculty of Pharmaceutical Sciences, Federal University of Amazonas (UFAM), Manaus, Brazil; ^3^ CIBA, Northwest Research and Transfer Center of the Province of Buenos Aires (UNNOBA), CIT NOBA (UNNOBA-UNSAdA-CONICET), Junin, Bs.As., Argentina

**Keywords:** natural products, antitumoral, antiangiogenic, new drugs, mechanisms of action

Natural products can be isolated from several biological sources, such as plants, fungi, animals, plant cell cultures, among others. Some of these compounds can reduce tumor growth and the spreading of tumor cells by blocking their migration or preventing blood vessel formation that contribute to the colonization of distant tissues. A high percentage of drugs derived from natural products are published, however, most of them fail to gain approval for treating cancer by the European Medicines Agency in Europe or national regulatory authorities of regional reference in the Americas. It has been shown that combination of natural products with conventional clinical chemotherapy and radiotherapy in antitumoral and antiangiogenic treatments reduced the toxicity of the therapy (Tuorkey, 2015; Braicu et al., 2017). The major challenge is the lack of scientific evidence to support their approbation and determine the if it is beneficial to be used as monotherapy or if the combination with conventional therapies. Their use and approval require research that defines that these natural products offer adequate therapeutic action, are less toxic or have a synergistic effect and reduce toxicity in combination with chemotherapy and radiotherapy in combination.

In this special issue, several articles and reviews discuss the beneficial use of natural products as antitumoral/antiangiogenic agents compared or in combination with synthetic conventional chemotherapy.

The original article by Liang and collaborators discusses the use of a flavonoid isolated from the rhizome of *Alpinia officinarum*: galangin. The authors used *in vitro* and *in vivo* gastric tumor models, demonstrating that this compound inhibited tumor growth by modulating different signals, such as p-JAK2, p-STAT3, Bcl-2, cleaved caspase-3, cleaved PARP, and Ki67. Specifically, galangin appears to induce apoptosis and decrease cell proliferation, by modulating STAT3/ROS axis, demonstrating the potential application of galangin for gastric cancer therapy.

Vitale and collaborators’ review provides an overview of 4-methylumbelliferone (4-MU), an orally available dietetic product, derivative of coumarin and mainly found in the plant family Umbelliferae or Apiaceae, focusing on its utility in different solid and hematological cancer. The authors discuss 4-MU mechanisms of action observed in tumors in different human models. They discuss different molecular mechanisms of 4-MU associated with its capacity to inhibit hyaluronan molecules in the tumor extracellular matrix and the biological impact on different cells of the tumor microenvironment. Finally, the authors comment about the possibility of 4-MU use as a co-adjuvant drug in conventional antineoplastic therapies. And since 4-MU, originally identified as a hepatoprotective component approved in European countries, could be considered its repositioning as an antitumor drug.

The original work of Zhang and collaborators discusses the role of Saikosaponin A (SSA), a main triterpenoid saponin component from *Radix bupleurum*. Their findings first revealed that SSA possesses potent antiangiogenic activities, thereby suppressing tumor growth by blocking VEGFR2 signaling pathways.

Wang and collaborators’ original article show a gut microbiota study in which they report the identification of one anticancer gut bacterial strain (AD16). Five new compounds were isolated and identified (streptonaphthalenes A and B (1–2), pestaloficins F and G (3–4), and eudesmanetetraiol A (5)), together with nine previously known compounds, were isolated from the effective fractions of AD16. The analysis of network pharmacology suggested that three compounds could be the key components for the anti-NSCLC (non-small cell lung cancer) activity of AD16. In addition to the PI3K–Akt signaling pathway, the proteoglycans in the cancer pathway could be involved in the anti-NSCLC action of AD16.

Yangbo and collaborators showed that the combination of sodium butyrate with cisplatin enhanced the apoptosis in gastrointestinal cancer cells through the mitochondrial apoptosis-related pathways *in vitro* and *in vivo*. Their combination produced a synergic effect. Sodium butyrate showed to be an alternative to other conventional chemotherapeutic drugs because it causes less cytotoxic effects, since it acts by modulating the intestinal microbiota, with studies already proving both its improvement the patient’s immune system and its effective use in other types of cancer. This, suggests that this combination may be an alternative for the treatment of a gastrointestinal cancer.

In another original paper, Liu and collaborators studied the effects of gracillin against gastric carcinoma cell line. They demonstrated that gracillin acted as inducer of the endogenous apoptosis, inhibiting cell migration and EMT (epithelial-mesenchymal transition process) pathway in BGC-823 cell line, through the tumor necrosis factor–α inducible protein-8, also called TIPE2. The EMT process is involved in tumor metastasis, characterized by high expression of N-cadherin and vimentin and low expression of E-cadherin. Thus, the authors demonstrated that gracillin has the potential to suppress tumor cell migration through the EMT process in the researched cell line, contributing to the emergence of another possible molecule to act against gastric cancer, currently the third most common cause of cancer in the world.

Zhan and collaborators reviewer the effects and mechanisms of OSW-1 (isolated from *Ornithogalum saundersiae*) against cancer *in vitro* and *in vivo*. OWS-1 was tested in the U.S National Cancer Institute in 60-cell lines *in vitro* screening panel and from these results its mechanism of action was explored. It has been shown to be cytotoxic against neoplastic cells of the ovary, breast, cervix, colon, leukemia, hepatocellular carcinoma and other cancer cells. *In vitro*, OSW-1 had activity on the inhibition of cell proliferation, acting to stop the cell cycle, inducing cell death by apoptosis and, at high concentrations, by necrosis and Golgi stress response. *In vivo* it was effective in inhibiting the growth of tumors such as breast cancer, colon cancer and leukemia. In general, it was able to inhibit tumor growth with a reduction in tumor size and weight, less metastatic nodules in the lungs and longer survival. In the case of the NFATc2 knockdown model, the NFATc2 may be related to suppression of migration and tumor invasion. The compound regulated the action on angiogenesis and the regulation of miRNA expression and various signaling pathways.

Cháirez-Ramírez et al. article reviews the role of the most studied polyphenols in the regulation of key elements of cancer signaling pathways and highlights the importance of a profound understanding of these regulations in order to improve cancer treatment and control with natural products.

Chang and collaborators reviewer the bibliographic background of herbal compounds from traditional Chinese medicine (TCM) applied for treating colorectal cancer (CRC). The authors focused their discussion on the Wnt/β-catenin signaling pathway since it plays a vital role in the initiation and progression of CRC. Also, how these natural compounds can be used in different stages of tumor disease, from precancerous lesions such as polyps, carcinoma *in situ* to metastatic cancer. Besides, they make an extensive comparison between TCM with small molecules or new drugs targeting Wnt/β-catenin used in combination with traditional chemotherapy that are under preclinical, clinical phase or FDA approved.

## References

[B1] Braicu C. Mehterov N. Vladimirov B. Sarafian V. Nabavi S. M. Atanasov A. G. (2017). Nutrigenomics in cancer: Revisiting the effects of natural compounds. Semin. Cancer Biol. 46, 84–106. 10.1016/j.semcancer.2017.06.011 28676460

[B2] Tuorkey M. J. (2015). Cancer therapy with phytochemicals: Present and future perspectives. Biomed. Environ. Sci. 28 (11), 808–819. 10.3967/bes2015.112 26695359

